# Nanotechnology in Skin Cancer Therapy: Recent Progress in Targeted Delivery

**DOI:** 10.1002/kjm2.70130

**Published:** 2025-10-23

**Authors:** Huang‐Ping Yu, Ching‐Yun Hsu, Jia‐You Fang, Zih‐Chan Lin

**Affiliations:** ^1^ Department of Anesthesiology Chang Gung Memorial Hospital at Linkou Taoyuan Taiwan; ^2^ School of Medicine, College of Medicine Chang Gung University Taoyuan Taiwan; ^3^ Department of Nutrition and Health Sciences Chang Gung University of Science and Technology Taoyuan Taiwan; ^4^ Research Center for Food and Cosmetic Safety and Center for Drug Research and Development Chang Gung University of Science and Technology Taoyuan Taiwan; ^5^ Pharmaceutics Laboratory, Graduate Institute of Natural Products Chang Gung University Taoyuan Taiwan; ^6^ Chronic Diseases and Health Promotion Research Center Chang Gung University of Science and Technology Chiayi Taiwan

**Keywords:** anticancer drug, drug targeting, nanocarrier, nanomedicine, skin cancer

## Abstract

Skin cancer, encompassing melanoma and non‐melanoma types, remains a significant public health concern globally. Conventional therapies—such as surgery, radiotherapy, chemotherapy, and immunotherapy—are constrained by poor skin penetration, systemic toxicity, and high recurrence rates. Nanotechnology has emerged as a promising strategy to address these limitations through enhanced drug delivery, targeted tumor accumulation, and reduced off‐target effects. This review summarizes recent advances in nanocarrier‐based approaches for skin cancer therapy. Key platforms include liposomes, polymeric nanoparticles, dendrimers, metallic nanoparticles, and biomimetic systems. These nanocarriers facilitate passive, active, and stimuli‐responsive targeting, thereby improving drug distribution within tumors and enhancing therapeutic precision. Applications include chemotherapy, photothermal and photodynamic therapy, gene and RNA delivery, and immunotherapy. Despite substantial preclinical success, challenges persist in translating findings to the clinic. These include limited dermal penetration, tumor heterogeneity, immune clearance, and regulatory barriers. Innovative solutions—such as multifunctional nanocarriers, personalized formulations, and non‐invasive delivery devices—are being investigated to address these issues. In conclusion, nanotechnology holds considerable potential to transform skin cancer treatment. Continued interdisciplinary efforts are crucial for translating laboratory innovations into clinically viable therapies, ensuring safer and more effective outcomes for patients.

## 1 Introduction

### 1.1 Skin Cancer Epidemiology

**FIGURE 1:**
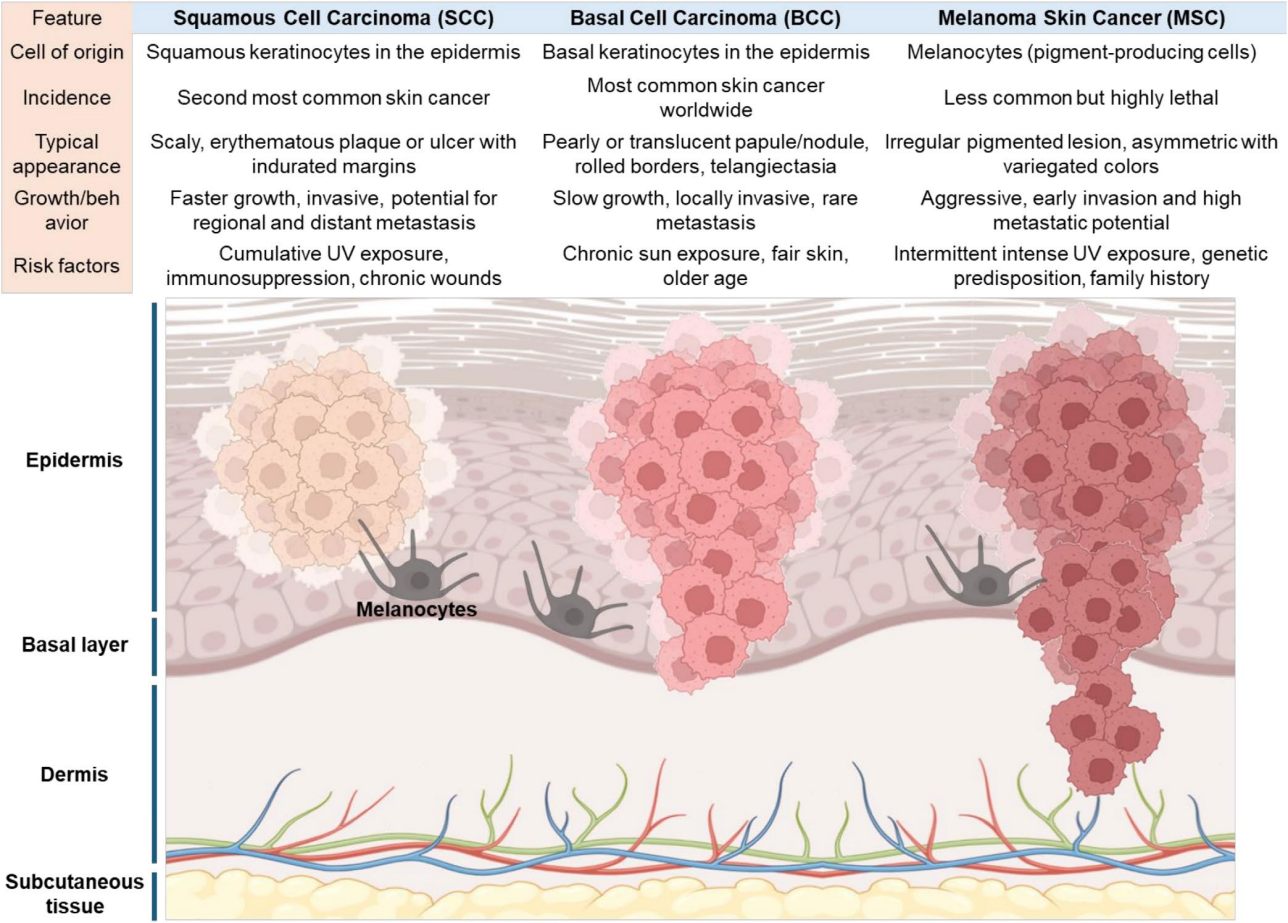
Comparative features of major skin cancers. Schematic representation of the three predominant types of skin cancer: squamous cell carcinoma (SCC), basal cell carcinoma (BCC), and melanoma skin cancer (MSC). The upper panel summarizes key characteristics, including cell of origin, incidence, clinical appearance, growth behavior, and risk factors. The lower illustration depicts their histological localization within the epidermis and dermis: SCC arises from keratinocytes in the squamous layer, BCC originates from basal keratinocytes, and MSC develops from melanocytes. While BCC and SCC are more common and generally less aggressive, melanoma carries a higher metastatic potential and mortality risk. Created in BioRender. https://BioRender.com/3fw90q0.

Skin cancer, comprising melanoma (MSC) and non‐melanoma skin cancers (NMSCs) such as basal cell carcinoma (BCC) and squamous cell carcinoma (SCC), represents a major global health burden with an increasing incidence across all regions (Figure 1). Although MSC accounts for a smaller proportion of skin cancers, it is more aggressive and associated with significantly higher mortality. According to GLOBOCAN 2020, there were over 324,000 new cases of melanoma and more than 57,000 related deaths, with the highest incidence observed in Europe and Oceania, particularly among fair‐skinned populations [1]. In contrast, NMSCs—especially BCC—are estimated to be 18–20 times more common than melanoma and are the most frequently diagnosed cancers among light‐skinned individuals [2, 3]. While BCC rarely metastasizes, SCC accounts for the majority of NMSC‐related deaths, particularly in elderly or immunocompromised patients [4]. Despite advances in early detection tools such as dermoscopy and AI‐assisted imaging, accurate differentiation between indolent and high‐risk lesions remains challenging [5]. Visual assessment is affected by diagnostic variability due to lesion heterogeneity, artifacts, and inconsistent imaging quality [6]. Moreover, current clinical practice continues to rely heavily on surgical excision or radiotherapy, which may be suboptimal in frail or elderly patients, and does not entirely prevent recurrence. Systemic therapies, although effective in advanced melanoma, often exhibit poor skin selectivity, limited penetration through the stratum corneum, and off‐target toxicities [7]. Nanotechnology‐based delivery systems have shown potential to overcome some of these limitations by enhancing localized drug accumulation and reducing systemic exposure. However, their translation to clinical practice is limited by difficulties in large‐scale manufacturing, batch consistency, long‐term biosafety, and regulatory barriers [8]. Furthermore, high costs and lack of access to precision diagnostics and targeted therapies in low‐resource settings contribute to persistent disparities in outcomes. Overall, while melanoma poses a higher per‐case fatality, the global disease burden of NMSCs—driven by prevalence, recurrence, and treatment costs—is substantially greater. Addressing both melanoma and NMSCs requires not only innovation in drug delivery and diagnostics but also the development of accessible, affordable, and scalable solutions tailored to regional epidemiological profiles and healthcare capacities.

### 1.2 Limitations of Conventional Therapies

Conventional modalities remain the backbone of skin cancer management, yet they face substantial limitations in advanced or recurrent disease. Surgical excision is the standard of care for most basal cell carcinoma (BCC), cutaneous squamous cell carcinoma (cSCC), and localized melanoma. While surgery offers high cure rates, it is often constrained by cosmetic and functional morbidity, particularly for lesions located on the face, scalp, or other anatomically sensitive sites [9]. Moreover, complete resection is difficult in locally advanced tumors with perineural invasion or bone involvement, leading to recurrence risks. Radiotherapy (RT) serves as an alternative or adjuvant modality, particularly for inoperable patients or those with high‐risk histological features. Despite its established efficacy, RT is restricted by dose‐limiting toxicities such as dermatitis, mucositis, and long‐term fibrosis, especially in the head and neck region. In melanoma, a historically radioresistant tumor, RT often provides only palliative benefits rather than durable tumor control. Technical challenges in achieving uniform dose distribution in cutaneous lesions can also limit therapeutic outcomes [10]. Chemotherapy, once a mainstay for metastatic melanoma and high‐risk cSCC, is now largely replaced by targeted and immune‐based therapies. Platinum‐based regimens or cisplatin‐doxorubicin combinations can induce partial responses, but their benefits are transient, with median progression‐free survival rarely exceeding 1 year. Severe systemic toxicities—including nephrotoxicity, myelosuppression, and neuropathy—further restrict their long‐term applicability [11]. Immunotherapy, particularly immune checkpoint inhibitors (anti‐PD‐1/PD‐L1 and anti‐CTLA‐4), has revolutionized outcomes for melanoma and advanced cSCC. Durable responses and survival benefits have been reported, with cemiplimab and pembrolizumab achieving objective response rates of 35%–50% in cSCC and melanoma [12]. However, challenges remain. A significant proportion of patients develop primary or acquired resistance, resulting in relapse after initial benefit. Additionally, immune‐related adverse events—including colitis, hepatitis, pneumonitis, and endocrinopathies—pose life‐threatening risks and limit widespread use. Importantly, immunotherapy efficacy is reduced in immunosuppressed patients (e.g., organ transplant recipients), who paradoxically represent a population at heightened risk for aggressive skin cancers [13]. Finally, across all systemic therapies, poor skin penetration and heterogeneous drug distribution within tumors remain challenges, particularly for topical or intralesional formulations. The cutaneous barrier restricts the delivery of chemotherapeutic and biologic agents to deep tumor nests, contributing to suboptimal responses. Collectively, the limitations of surgery, radiotherapy, chemotherapy, and immunotherapy highlight the urgent need for innovative strategies. Nanotechnology‐based targeted delivery platforms have thus emerged as promising tools to address systemic toxicity, drug resistance, and poor tissue penetration, paving the way for more effective and patient‐tailored skin cancer therapies.

### 1.3 Rationale for Nanotechnology in Skin Cancer Therapy

Conventional modalities for skin cancer treatment, including surgery, chemotherapy, radiotherapy, and immunotherapy, though clinically effective in certain stages, are frequently limited by systemic toxicity, poor patient compliance, and restricted therapeutic selectivity. Nanotechnology‐based strategies have emerged as a promising alternative, offering targeted delivery, improved drug bioavailability, and reduced off‐target effects, thereby addressing key shortcomings of conventional therapies [14]. One central rationale for the application of nanotechnology in oncology is its ability to deliver targeted treatment. Nanoparticles employ both passive and active targeting mechanisms to achieve selective accumulation within tumors. Passive targeting relies on the enhanced permeability and retention (EPR) effect, whereby the leaky vasculature and impaired lymphatic drainage of tumor tissue facilitate preferential nanoparticle accumulation [15]. Active targeting further refines this process by functionalizing nanocarrier surfaces with ligands such as antibodies, peptides, or sugars, enabling receptor‐mediated uptake by malignant cells. Such strategies enhance therapeutic precision while sparing normal skin cells, a critical advantage given the delicate architecture and high regenerative turnover of the skin [16].

A second rationale is improved bioavailability. Many anticancer agents, particularly hydrophobic compounds such as curcumin, resveratrol, or itraconazole, exhibit poor solubility and instability when administered via conventional routes. Encapsulation within nanosystems—including polymeric nanoparticles, liposomes, solid lipid nanoparticles, and hybrid carriers—protects labile drugs from premature degradation, increases aqueous solubility, and provides sustained release profiles. Moreover, topical nanocarrier formulations overcome the barrier posed by the stratum corneum, enhancing dermal penetration and local retention, thereby achieving therapeutically relevant concentrations at the tumor site. Such approaches allow dose reduction while maintaining efficacy, minimizing systemic exposure and adverse effects [17, 18].

A third rationale is the reduction of off‐target effects, which remain a major limitation of systemic chemotherapy and radiotherapy. By localizing drug release to malignant tissues, nanocarriers minimize damage to healthy keratinocytes, fibroblasts, and melanocytes. Surface modifications, such as PEGylation, prevent nonspecific uptake and prolong circulation, while stimuli‐responsive designs enable drug release in response to tumor‐specific triggers such as pH, enzymes, or oxidative stress. In photodynamic therapy (PDT), for example, nanocarriers improve the delivery of photosensitizers into tumor tissue, reducing photosensitivity and systemic toxicity while maximizing reactive oxygen species generation upon irradiation. Similarly, dual drug‐loaded liposomal systems achieve synergistic effects by co‐delivering chemotherapeutics with anti‐inflammatory or immunomodulatory agents, thereby enhancing antitumor efficacy while minimizing collateral toxicity [19, 20, 21].

Notably, nanosystems not only act as passive carriers but may also possess intrinsic therapeutic or synergistic properties. Certain metallic nanoparticles exhibit photothermal or cytotoxic effects, and lipid‐polymer hybrids can modulate immune responses, further expanding the therapeutic arsenal against skin cancers. Collectively, these advantages underscore the potential of nanotechnology in skin cancer therapy, positioning it as a foundation for next‐generation interventions that aim to improve treatment efficacy, safety, and patient outcomes.

## 2 Nanotechnology Platforms for Skin Cancer Therapy

### 2.1 Overview of Nanocarriers

**FIGURE 2:**
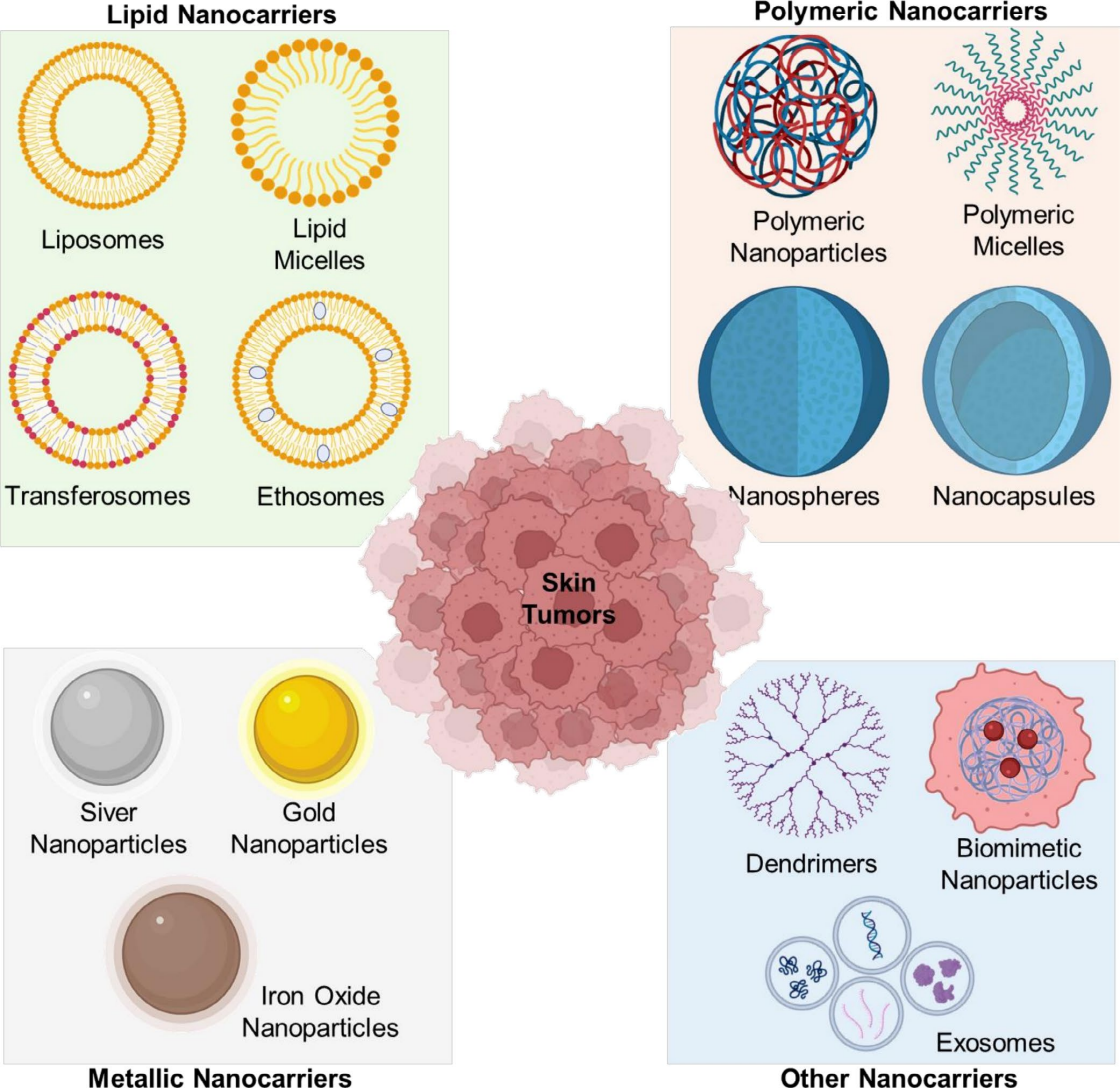
Nanocarrier platforms for targeted skin cancer therapy. Schematic overview of representative nanocarriers explored in cutaneous oncology. Lipid‐based systems (liposomes, lipid micelles, transferosomes, ethosomes), polymeric nanoparticles (polymeric micelles, nanospheres, nanocapsules), and metallic nanoparticles (silver, gold, and iron oxide) have been widely utilized for drug and gene delivery. In addition, dendrimers, biomimetic nanoparticles, and exosomes provide versatile strategies for tumor targeting and modulation of the tumor microenvironment. These diverse nanoplatforms enable improved skin penetration, controlled release, and enhanced therapeutic efficacy in skin tumors. Created in BioRender. https://BioRender.com/mzc1fat.

Nanotechnology has enabled the development of diverse nanocarriers tailored for skin cancer therapy, each offering unique physicochemical properties that improve therapeutic delivery, enhance drug stability, and minimize systemic toxicity. Among the most widely studied systems are liposomes, polymeric nanoparticles, dendrimers, metallic nanoparticles, and emerging hybrid or biomimetic carriers (Figure 2). Liposomes are spherical vesicles composed of phospholipid bilayers surrounding an aqueous core, capable of encapsulating both hydrophilic and hydrophobic agents. Their biocompatibility and versatility have established them as a benchmark nanocarrier in the field of oncology. In melanoma models, peptide‐modified liposomes demonstrated enhanced skin penetration and selective cytotoxicity while reducing systemic toxicity compared with oral or intravenous formulations. However, their relatively rigid bilayer and limited penetration depth restrict efficiency, leading to the development of deformable vesicles such as transferosomes and ethosomes [22, 23, 24].

Polymeric nanoparticles (PNPs), including nanospheres, nanocapsules, and micelles, are fabricated from biodegradable polymers such as PLGA, PEG, or chitosan. They provide controlled drug release, protection against enzymatic degradation, and flexibility for surface modification. Polymeric micelles functionalized with folic acid or other ligands have been shown to selectively enhance uptake by melanoma cells, reduce tumor burden, and overcome multidrug resistance. Furthermore, nanosponges and nanocapsules can encapsulate both hydrophobic and hydrophilic drugs, improving solubility and extending circulation times [25, 26, 27].

Dendrimers represent another class of polymeric carriers distinguished by their branched, tree‐like architecture and tunable surface functionalities. Their multivalent surface allows conjugation with targeting ligands, imaging agents, or therapeutic molecules. Dendrimer‐based radionuclide conjugates, such as ^188Re‐loaded dendrimers, have shown significant antitumor efficacy in melanoma models, underscoring their potential in theranostic applications [28, 29, 30].

Metallic nanoparticles (MNPs), particularly gold, silver, and iron oxide nanoparticles, have emerged as potent therapeutic and diagnostic platforms. Their unique optical, magnetic, and catalytic properties enable multifunctional use, including photothermal ablation, radiosensitization, and targeted drug delivery. Gold nanoparticles (AuNPs) conjugated with chemotherapeutics or antibodies have induced selective apoptosis in melanoma cells through ROS generation and mitochondrial dysfunction. Similarly, silver nanoparticles exhibit strong cytotoxicity against melanoma by enhancing ROS and triggering apoptosis, while iron oxide nanoparticles can be applied in magnetic hyperthermia and MRI‐guided therapy. Despite their advantages, concerns remain regarding long‐term toxicity, biodistribution, and environmental impact [31, 32, 33].

Hybrid and biomimetic systems are designed to combine the benefits of synthetic nanocarriers with the biological functions of natural vesicles. Exosomes, naturally secreted nanosized vesicles, inherently carry proteins and nucleic acids that facilitate intercellular communication, making them highly biocompatible delivery vehicles. Engineered exosomes loaded with anticancer agents have demonstrated efficient tumor targeting while evading immune clearance. Similarly, cell membrane–coated nanoparticles, cloaked with membranes derived from erythrocytes, platelets, or cancer cells, provide immune evasion, prolonged circulation, and homotypic tumor targeting. These biomimetic systems represent a next generation of nanocarriers bridging synthetic precision with biological functionality [34, 35, 36].

Collectively, these nanocarriers highlight the diversity of design strategies available to address the multifaceted challenges of skin cancer therapy. Liposomes and polymeric nanoparticles remain foundational due to their safety and versatility; dendrimers provide precise multivalent targeting; metallic nanoparticles offer diagnostic and therapeutic multifunctionality; and hybrid/biomimetic systems provide enhanced physiological integration. Continued optimization of these platforms—focusing on stability, targeted delivery, and clinical scalability—will be essential for translating nanocarrier‐based therapies into routine management of skin cancers. To provide a comprehensive overview, a table summarizing the diversity of nanocarrier platforms and their therapeutic applications in skin cancer was compiled (Table 1).

**TABLE 1:** Comparison of major nanocarrier types for skin cancer therapy: key examples, advantages, and limitations.

Nanocarrier type	Key examples	Advantages	Limitations
Lipid‐based NPs	Liposomes, solid lipid nanoparticles (SLNs), nanostructured lipid carriers (NLCs)	High biocompatibility, encapsulate hydrophilic/lipophilic drugs, enhance skin penetration	Stability issues, low drug loading in some systems
Polymeric NPs	PLGA, chitosan, dendrimers	Biodegradable, tunable release, surface functionalization possible	Potential immunogenicity (dendrimers), scale‐up challenges
Metallic NPs	Gold, silver, iron oxide	Photothermal effect, imaging capability, multifunctional design	Risk of toxicity, long‐term accumulation
Hybrid/Biomimetic	Exosome‐mimetic vesicles, peptide‐modified NPs	Natural targeting ability, immune evasion	Complex preparation, cost

### 2.2 Advantages in Skin Delivery

The formidable barrier properties of the stratum corneum limit the effective delivery of therapeutic agents into deeper skin layers, posing significant challenges for skin cancer treatment. Nanotechnology offers innovative approaches to overcome these limitations by enhancing drug penetration, accumulation, and controlled release.

#### 2.2.1 Enhanced Skin Penetration and Accumulation

Traditional formulations often fail to deliver drugs beyond the superficial epidermis because of the lipophilic yet tightly packed structure of the stratum corneum. Nanocarriers, including liposomes, polymeric nanoparticles, dendrimers, and lipid‐based vesicles, can circumvent this barrier by exploiting their small size, surface charge, and deformability. Studies have demonstrated that nanocarriers < 100 nm in diameter with amphiphilic properties efficiently intercalate into intercellular lipid domains and accumulate within skin layers. In addition, follicular and appendageal pathways act as secondary “gateways” that nanocarriers can exploit, enhancing the local retention of anticancer agents. The incorporation of chemical enhancers (e.g., fatty acids, terpenes) or physical strategies such as microneedles further improves penetration by creating transient channels that facilitate deeper drug transport. Compared to conventional formulations, nanocarriers reduce systemic exposure, prolong dermal residence time, and increase drug concentration at tumor sites, thereby enhancing therapeutic efficacy while minimizing off‐target effects [37, 38, 39].

#### 2.2.2 Controlled Release

Beyond penetration, sustained and localized drug availability is crucial for maximizing therapeutic outcomes in skin cancer. Burst release from conventional vehicles often results in irritation or systemic toxicity, whereas nanocarriers provide the advantage of controlled and extended release. For instance, dendrimers functionalized with biocompatible polymers can encapsulate hydrophobic drugs and release them gradually under skin‐relevant conditions (pH 5–6, 37°C), ensuring prolonged therapeutic action with reduced dosing frequency. Lipid‐based carriers, such as ethosomes and transferosomes, not only enhance permeation but also sustain release within the epidermis and dermis, maintaining drug levels over extended periods. This extended‐release behavior reduces dosing intervals, improves patient compliance, and maintains consistent drug exposure, which is particularly beneficial in chronic regimens for melanoma or non‐melanoma skin cancers [8, 40, 41].

#### 2.2.3 Stimuli‐Responsive Release

The tumor and inflamed skin microenvironment is characterized by acidic pH, elevated reactive oxygen species (ROS), enzymatic activity, and hypoxia. Stimuli‐responsive nanocarriers utilize these hallmarks to enable spatiotemporally controlled release, thereby enhancing precision and minimizing collateral damage [42]. pH‐sensitive dendrimers or liposomes remain stable at physiological pH but rapidly release their payload under acidic tumor conditions [43]. Similarly, ROS‐responsive systems containing thioketal linkages degrade in oxidative environments, triggering on‐demand drug release. Multi‐stimuli responsive designs integrate endogenous (pH, enzymes, ROS) and exogenous (light, heat, ultrasound) triggers to achieve sequential penetration, accumulation, and intracellular release [44]. Such systems selectively activate therapeutic payloads at malignant or inflamed sites while sparing normal tissues, offering a promising strategy to address heterogeneity in tumor microenvironments. Notably, in cutaneous cancers, externally applied stimuli such as near‐infrared light can be conveniently harnessed for localized activation, enhancing precision.

#### 2.2.4 Clinical Relevance

Collectively, these advances address critical bottlenecks in skin cancer therapy. Enhanced penetration ensures that therapeutic agents reach malignant cells within the dermis; controlled release maintains drug concentrations for sustained cytotoxicity; and stimuli‐responsiveness aligns drug activation with tumor‐specific microenvironmental cues. These features reduce systemic toxicity, mitigate drug resistance, and improve patient adherence. Moreover, by co‐delivering small molecules, peptides, or genetic cargos, nanocarriers provide versatile therapeutic options ranging from chemotherapeutics to immunomodulators. Although challenges such as large‐scale reproducibility and long‐term safety remain, the integration of nanocarrier‐based delivery with precision medicine approaches holds promise for transforming the management of skin cancer [45, 46].

## 3 Targeting Strategies for Nanoparticle‐Based Delivery

### 3.1 Passive Targeting

Passive targeting exploits the enhanced permeability and retention (EPR) effect within tumor tissues. Cutaneous malignancies often exhibit leaky vasculature, impaired lymphatic drainage, and inflamed stroma, which allow nanoscale carriers to accumulate preferentially at the diseased site. Studies have demonstrated that nanoparticles smaller than 100 nm readily penetrate the dermis and are retained within tumor microenvironments, thereby extending drug residence and improving local concentration. This effect has been particularly advantageous for polymeric and lipid nanocarriers, which prolong circulation time and enable controlled release once localized, thereby reducing systemic exposure and toxicity [47, 48].

### 3.2 Active Targeting

To further enhance specificity, active targeting strategies modify nanoparticle surfaces with ligands that recognize overexpressed receptors on cancer cells. Antibodies, peptides, folate, and hyaluronic acid have been widely investigated as conjugates. For example, folic acid–modified carriers show selective uptake by squamous cell carcinoma, where folate receptors are abundant. Similarly, hyaluronic acid–decorated systems exploit the CD44 receptor, a marker frequently upregulated in melanoma and keratinocyte carcinomas [49]. Receptor‐mediated delivery has also been achieved using ligands for epidermal growth factor receptor (EGFR) or integrins such as αvβ3, which mediate tumor invasion [50]. Titanium dioxide nanoparticles conjugated with an RGD peptide exhibited selective binding to integrin‐expressing melanoma cells, enhancing photodynamic cytotoxicity [51]. Similarly, aptamer‐functionalized lipid–polymer hybrids demonstrated improved uptake in CD20‐positive melanoma cells, producing superior tumor suppression compared to unconjugated formulations [52]. Beyond these ligand‐based approaches, recent advances have introduced dual cell‐penetrating peptide–conjugated nanocarriers, in which R9 and p28 peptides are anchored onto PLGA nanoparticles to deliver miR‐205‐5p into cutaneous squamous cell carcinoma. This dual‐peptide design significantly enhanced cellular uptake and tumor penetration, resulting in stronger antiproliferative and pro‐apoptotic effects than single‐peptide or naked miRNA systems, underscoring the potential of CPP‐mediated active targeting in skin cancer therapy [53]. These examples highlight how rational surface engineering augments tumor selectivity and therapeutic potency.

### 3.3 Stimuli‐Responsive Systems

Beyond passive and ligand‐based strategies, stimuli‐responsive designs enable site‐specific drug release triggered by internal or external cues. The acidic milieu of tumor tissues creates a natural pH gradient that destabilizes pH‐sensitive polymers, leading to targeted payload liberation. Redox‐responsive nanoparticles, often incorporating disulfide linkages, undergo cleavage in glutathione‐rich tumor cytosol, ensuring intracellular release. Enzyme‐sensitive carriers that degrade upon contact with matrix metalloproteinases abundant in tumor stroma further exemplify endogenous responsiveness [54, 55]. Exogenous stimuli such as light or magnetic fields have also been applied in localized skin cancer therapy. Gold and silver nanoparticles exhibit strong plasmonic absorption, enabling photothermal ablation of tumor cells under near‐infrared irradiation. In photodynamic applications, nanoparticle‐encapsulated photosensitizers generate reactive oxygen species only upon illumination, enhancing selectivity and minimizing collateral damage [31]. Magnetic nanoparticles, on the other hand, can be guided to lesion sites and activated by alternating magnetic fields, providing both therapeutic heating and imaging capabilities [56]. These multifunctional systems enable spatiotemporal control of drug action, offering a promising avenue for non‐invasive, skin‐localized interventions. In skin cancer therapy, nanoparticle targeting strategies include passive accumulation via the EPR effect, active ligand‐mediated recognition of tumor receptors, and stimuli‐responsive systems that release drugs in response to tumor‐specific or externally applied triggers. Collectively, these approaches maximize therapeutic efficacy, reduce systemic toxicity, and provide precision control, establishing nanotechnology as a transformative modality for dermatologic oncology.

## 4 Recent Progress in Therapeutic Applications

### 4.1 Chemotherapy‐Loaded Nanocarriers

Conventional chemotherapeutics such as doxorubicin, paclitaxel, and cisplatin are widely used against skin cancers; however, systemic toxicity and poor tumor accumulation limit their efficacy. Nanocarriers have been developed to improve drug solubility, stability, and site‐specific delivery. Doxorubicin‐loaded cationic lipid nanoparticles combined with iontophoresis showed enhanced penetration and cytotoxicity in skin tumor models. Similarly, liposomal cisplatin formulations (e.g., NC‐6004, SPI‐77) and polymeric micelles demonstrated improved pharmacokinetics and reduced nephrotoxicity [57, 58]. For paclitaxel, protein‐bound nanoparticles such as Abraxane and liposome‐entrapped paclitaxel (LEP‐ETU) formulations have reached clinical trials, underscoring their translational promise [59]. Collectively, these systems reduce off‐target damage and allow higher intratumoral accumulation, thereby improving therapeutic outcomes in cutaneous malignancies.

### 4.2 Photothermal and Photodynamic Therapy

**FIGURE 3:**
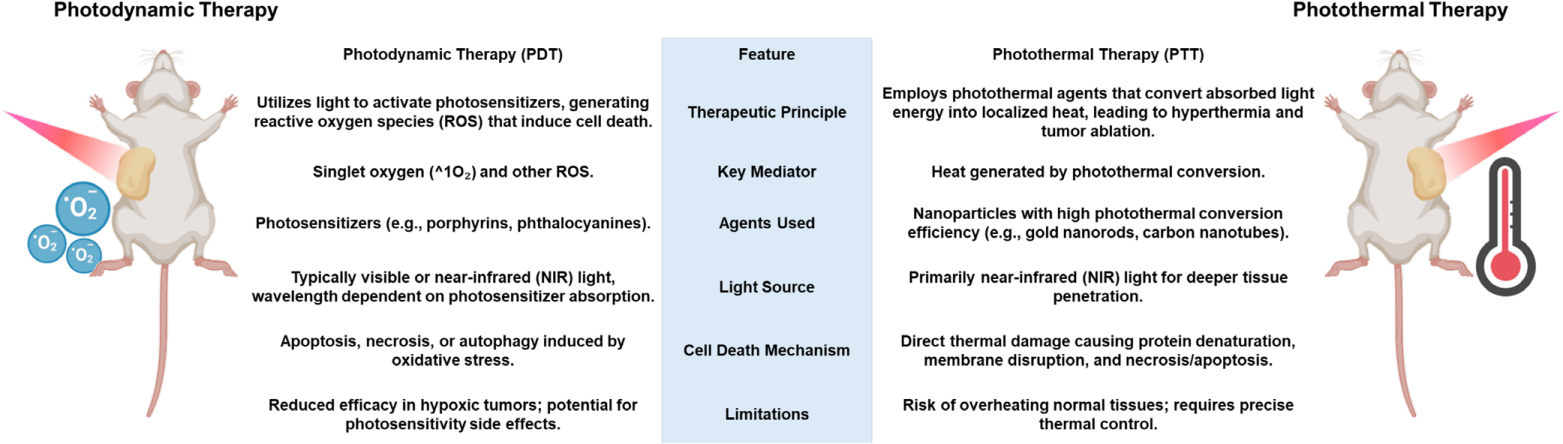
Comparative overview of photodynamic therapy (PDT) and photothermal therapy (PTT) in skin cancer treatment. Schematic illustration comparing PDT and PTT in murine tumor models. PDT relies on photosensitizers activated by specific light wavelengths to generate reactive oxygen species (ROS), leading to oxidative tumor cell death. In contrast, PTT employs photothermal agents that convert light energy into localized heat, inducing thermal ablation of cancer cells. The accompanying table summarizes their therapeutic principles, key mediators, agents used, light sources, mechanisms of cell death, and limitations. Together, these modalities represent complementary nanotechnology‐enabled strategies for skin cancer therapy. Created in BioRender. https://BioRender.com/zgf49kw.

Photothermal therapy (PTT) and photodynamic therapy (PDT) have gained increasing attention in melanoma and nonmelanoma skin cancers (Figure 3). Gold nanoshells and nanorods exhibit strong absorption in the near‐infrared region, enabling localized heating and tumor ablation. Carbon‐based nanostructures, such as single‐walled carbon nanotubes, provide both photothermal conversion and drug‐loading capacity, supporting multimodal therapy [60, 61]. In PDT, photosensitizers encapsulated in liposomes, micelles, or polymeric nanoparticles overcome hydrophobicity and stability limitations [62]. For instance, protoporphyrin IX‐loaded PLGA nanoparticles and verteporfin‐loaded silica carriers improved melanoma inhibition in vitro and in vivo [63]. Combination photothermal–photodynamic approaches, such as zinc phthalocyanine‐gold nanorod conjugates, further enhance ROS generation and hyperthermic destruction of melanoma cells [64, 65].

### 4.3 Gene and RNA‐Based Therapy

Nanocarriers also facilitate gene modulation strategies for treating skin cancer. Delivery of siRNA and miRNA remains challenging due to nuclease degradation and poor cellular uptake, but lipid‐based nanoparticles, cyclodextrin polymers, and polymeric assemblies now enable efficient intracellular release [66]. For example, esterase‐responsive charge‐reversal polymers have been used to deliver pro‐apoptotic genes selectively in tumor cells, achieving strong therapeutic responses. CRISPR/Cas systems are also under exploration, with silica and magnetic nanoparticles being developed to encapsulate nucleic acids and support tumor‐specific genome editing [67]. These advances highlight the potential of nanotechnology to achieve precise genetic interventions in cutaneous oncology.

### 4.4 Immunotherapy and Vaccine Delivery

Nanoparticles are emerging as powerful tools to modulate anti‐tumor immunity. They can serve as adjuvants or carriers for antigens, checkpoint inhibitors, and cytokines. For instance, magnetic nanoparticles conjugated with tumor antigens have been shown to enhance antigen presentation and T‐cell activation, supporting their use in cancer vaccines [68]. Liposomes and polymeric systems have also been used to co‐deliver tumor antigens and immunostimulatory molecules, thereby improving immune recognition while reducing systemic inflammation [69]. Additionally, nanoparticle‐based laser immunotherapy, which combines photothermal ablation with immune stimulation, demonstrated synergistic eradication of melanoma [70]. In summary, recent advances in chemotherapy‐loaded nanocarriers, photothermal and photodynamic systems, genetic payload delivery, and immunomodulatory nanoparticles highlight the versatility of nanotechnology in skin cancer therapy. These approaches not only enhance drug delivery and efficacy but also create new opportunities for combinatorial and personalized treatments. Table 2 summarizes selected therapeutic strategies that have been evaluated in preclinical and early clinical contexts.

**TABLE 2:** Nanoparticle‐based therapeutic strategies in skin cancer: Representative systems, cargos, and observed effects.

Therapeutic Strategy	Nanoparticle system	Cargo	Observed effect
Chemotherapy	Gold NPs, PLGA NPs	5‐FU, paclitaxel, doxorubicin	Enhanced tumor penetration, reduced systemic toxicity
Photodynamic/photothermal	Liposomes, silica NPs, gold nanoshells	ALA, photosensitizers	Improved light‐triggered tumor ablation
Gene/RNA delivery	Cationic gold NPs, mesoporous silica	siRNA, plasmid DNA	Downregulation of oncogenes (e.g., EGFR, Bcl‐2)
Immunotherapy/vaccines	Iron oxide NPs, lipid carriers	Peptide antigens, adjuvants	Potentiated antitumor immunity, vaccine transport

## 5 Challenges and Future Perspectives

Despite promising preclinical outcomes, the clinical translation of nanotechnology in skin cancer therapy remains limited by several barriers. The primary among these is the formidable stratum corneum, which restricts nanoparticle penetration into deeper skin layers and hinders consistent drug accumulation at tumor sites [71]. While modifications such as hyaluronate‐coating on solid lipid nanoparticles have shown improved penetration and tumor targeting, heterogeneity within skin tumors—both genetic and microenvironmental—creates variable responses and reduces the predictability of therapeutic efficacy [72]. Furthermore, nanoparticles are subject to rapid immune recognition and clearance, particularly by macrophages, which can shorten systemic circulation and therapeutic half‐life [73]. Balancing nanoparticle stability, immune evasion, and efficient tumor localization remains a persistent challenge [74]. Emerging strategies aim to overcome these challenges by combining complementary therapeutic modalities. Multifunctional nanocarriers designed to co‐deliver chemotherapeutics, photosensitizers, or immune modulators produce synergistic effects, enhancing tumor eradication while minimizing off‐target toxicity [75]. For instance, the integration of siRNA‐mediated VEGF silencing with nanoparticle‐enabled transdermal delivery has demonstrated significant inhibition of melanoma angiogenesis [76]. Similarly, RNA‐loaded nanoparticles capable of reprogramming immune responses, such as lipid nanoparticles carrying anti‐CD3 mRNA, illustrate the potential of nanomedicine in “heating up” immunologically cold tumors [77]. These advances indicate a growing shift toward combination therapies, with nanocarriers serving as central platforms for multimodal treatment.

Personalized nanomedicine represents another transformative direction. The ability to engineer nanoparticles with tumor‐specific ligands or patient‐tailored RNA payloads addresses the heterogeneity of skin cancers and opens pathways to individualized treatment regimens [78]. Coupled with non‐invasive topical or microneedle‐based delivery devices, these approaches could enable localized, patient‐friendly therapies with reduced systemic toxicity [79]. Notably, the development of transdermal nanocarrier systems demonstrates that effective treatment may be achieved without systemic exposure, offering safer and more accessible interventions for skin malignancies.

Moving forward, the integration of nanotechnology into clinical practice will depend on addressing the issues of scalability, reproducibility, and adherence to regulatory standards. Lessons from recent FDA approvals of siRNA and mRNA therapeutics underscore the importance of rigorous evaluation of nanoparticle safety, biodistribution, and long‐term effects [80]. With continued innovation in multifunctional design, patient‐specific platforms, and topical delivery devices, nanomedicine holds strong potential in reshaping the therapeutic landscape of skin cancer [81]. The outlook is optimistic: as translational challenges are progressively addressed, next‐generation nanocarriers may soon gain regulatory approval, providing clinicians with powerful, targeted tools to manage both melanoma and non‐melanoma skin cancers.

## 6 Conclusion

Nanotechnology has significantly advanced the field of skin cancer therapy by introducing diverse nanocarriers and targeting strategies that enhance drug solubility, improve skin penetration, and enable controlled release at tumor sites. Recent progress includes chemotherapy‐loaded systems, photothermal and photodynamic agents, gene‐ and RNA‐based approaches, and immunomodulatory nanoplatforms, each addressing the limitations of conventional modalities. Together, these innovations demonstrate the potential of nanomedicine to achieve higher therapeutic selectivity with reduced systemic toxicity. Despite encouraging preclinical results, the translation of nanoparticle‐based therapies into routine dermatologic oncology remains limited. Critical challenges include achieving consistent skin penetration, addressing tumor heterogeneity, ensuring large‐scale reproducibility, and addressing regulatory and biosafety concerns. Nevertheless, the approval of other nanoparticle formulations in oncology underscores the feasibility of adapting similar approaches to skin cancer care. In particular, the integration of stimuli‐responsive systems, personalized nanomedicine, and minimally invasive delivery devices indicates a clear path toward clinical applicability. Moving forward, bridging the gap between the laboratory and the clinic will require concerted efforts across disciplines. Materials scientists, dermatologists, oncologists, pharmacologists, and regulatory experts must collaborate to refine nanoparticle design, validate safety, and establish standardized manufacturing protocols. Equally important are translational studies and clinical trials that assess efficacy in diverse patient populations. By fostering multidisciplinary partnerships, targeted nanomedicine has strong potential to reshape skin cancer management, transforming therapeutic outcomes and providing safer, more effective options for patients worldwide.

## Conflicts of Interest

The authors declare no conflicts of interest.

## Data Availability

The data that support the findings of this study are available from the corresponding author upon reasonable request.
